# Molecular Behavior of α-Synuclein Is Associated with Membrane Transport, Lipid Metabolism, and Ubiquitin–Proteasome Pathways in Lewy Body Disease

**DOI:** 10.3390/ijms25052676

**Published:** 2024-02-26

**Authors:** Tomoya Kon, Seojin Lee, Ivan Martinez-Valbuena, Koji Yoshida, Satoshi Tanikawa, Anthony E. Lang, Gabor G. Kovacs

**Affiliations:** 1 Tanz Centre for Research in Neurodegenerative Disease, University of Toronto, 60 Leonard Ave., Toronto, ON M5T 0S8, Canada; tomoya.kon@mail.utoronto.ca (T.K.); lseojin.lee@mail.utoronto.ca (S.L.); ivan.martinez@utoronto.ca (I.M.-V.); koji.yoshida@mail.utoronto.ca (K.Y.); satoshi.tanikawa@utoronto.ca (S.T.); anthony.lang@uhn.ca (A.E.L.); 2 Department of Neurology, Hirosaki University Graduate School of Medicine, 5 Zaifu, Hirosaki 036-8562, Japan; 3 Department of Laboratory Medicine and Pathobiology, University of Toronto, 200 Elizabeth St., Toronto, ON M5G 2C4, Canada; 4 Department of Legal Medicine, Faculty of Medicine, University of Toyama, 2630 Sugitani, Toyama 930-0194, Japan; 5 Edmond J Safra Program in Parkinson’s Disease and Rossy Progressive Supranuclear Palsy Centre, Toronto Western Hospital, 399 Bathurst St., Toronto, ON M5T 2S8, Canada; 6 Laboratory Medicine Program, University Health Network, 200 Elizabeth St., Toronto, ON M5G 2C4, Canada; 7 Krembil Brain Institute, University of Toronto, 60 Leonard Ave., Toronto, ON M5T 0S8, Canada

**Keywords:** Lewy body disease, Parkinson’s disease, alpha-synuclein, nanoString, mRNA, transcriptome, seeding capacity, transmembrane transporter, lipid metabolism, ubiquitin

## Abstract

Lewy body diseases (LBDs) feature α-synuclein (α-syn)-containing Lewy bodies, with misfolded α-syn potentially propagating as seeds. Using a seeding amplification assay, we previously reported distinct α-syn seeding in LBD cases based on the area under seeding curves. This study revealed that LBD cases showing different α-syn seeding kinetics have distinct proteomics profiles, emphasizing disruptions in mitochondria and lipid metabolism in high-seeder cases. Though the mechanisms underlying LBD development are intricate, the factors influencing α-syn seeding activity remain elusive. To address this and complement our previous findings, we conducted targeted transcriptome analyses in the substantia nigra using the nanoString nCounter assay together with histopathological evaluations in high (n = 4) and low (n = 3) nigral α-syn seeders. Neuropathological findings (particularly the substantia nigra) were consistent between these groups and were characterized by neocortical LBD associated with Alzheimer’s disease neuropathologic change. Among the 1811 genes assessed, we identified the top 20 upregulated and downregulated genes and pathways in α-syn high seeders compared with low seeders. Notably, alterations were observed in genes and pathways related to transmembrane transporters, lipid metabolism, and the ubiquitin–proteasome system in the high α-syn seeders. In conclusion, our findings suggest that the molecular behavior of α-syn is the driving force in the neurodegenerative process affecting the substantia nigra through these identified pathways. These insights highlight their potential as therapeutic targets for attenuating LBD progression.

## 1. Introduction

Lewy body diseases (LBDs) are characterized by the presence of Lewy bodies (LBs), primarily composed of misfolded α-synuclein (α-syn), distributed extensively in the central and peripheral nervous systems [1,2,3,4]. The distribution of LBs defines various LBD phenotypes, including Parkinson’s disease (PD), PD with dementia (PDD), and dementia with Lewy bodies (DLBs) [1,2,3,4]. Clinical and pathological heterogeneities in LBD are believed to stem from molecular diversities (strains or polymorphs) of α-syn [2,4,5]. α-Syn exists in a natively unfolded monomeric form or a membrane-bound α-helical tetramer, with an amyloidogenic nature [2,4,5]. A conformational change, termed misfolding, induces the formation of aggregation nuclei, termed seeds, which self-propagate and spread between brain regions via cell-to-cell transmission mechanisms [2,4,5,6].

The seeding amplification assay (SAA) is a protein kinetics assay that has been widely employed to study the seeding activity of various amyloidogenic and disease-associated proteins, including α-syn. Elevated seeding activity likely plays a significant role in the propagation of misfolded α-syn [7]. We recently investigated α-syn seeding activity in synucleinopathies [8,9]. We categorized LBD patients as high, intermediate, or low seeders based on their α-syn seeding kinetics, demonstrating the influence of distinctly different α-syn kinetics on LBD clinical subtypes [8]. Proteomic analysis further indicated notable enrichment in membrane structure and significant disruptions in mitochondria and lipid metabolism in α-syn high seeders compared with low seeders [8]. A multifactorial mechanism involving dysfunction with membrane and intracellular trafficking, mitochondria, ubiquitin–proteasome system (UPS), autophagy–lysosomal pathway (ALP), lipid and vitamin metabolism, cytosolic Ca^2+^, axonal transport, synaptic transmission, neuroinflammation, post-translational protein modification, chromatin remodeling and apoptosis, the Wnt signaling pathway, and the Notch signaling pathway has been proposed for the development of LBD [10,11,12,13,14,15,16,17,18,19,20]. However, the factors influencing, and pathways associated with α-syn seeding activity remain elusive. To comprehensively understand the molecular signatures and mechanisms associated with α-syn seeding activity, we combined neuropathologic assessment and nanoString nCounter technology to investigate transcriptomic expression differences between α-syn high and low seeders in the substantia nigra (SN) in LBD.

## 2. Results

### 2.1. Definition of High and Low α-Syn Seeders

As we previously documented [8], neuropathological evaluation is not enough for classifying α-syn molecular behavior. In this study, we classified patients as high and low α-syn seeders by evaluating the area under the curve obtained after testing, using α-syn SAA, a cohort of 32 LBD patients using protein homogenates derived from the SN of these patients [8]. For this study, we selected four of the cases exhibiting the highest AUC values (that were designated as high seeders), while the three cases with the lowest AUC values were categorized as low seeders.

### 2.2. Demographics of Patients

The age at death, sex, disease duration, post-mortem interval, Braak LBD stage, and DV200 showed no significant differences between α-syn high and low seeders (Table 1).

### 2.3. Neuropathological Findings

**Figure 1:**
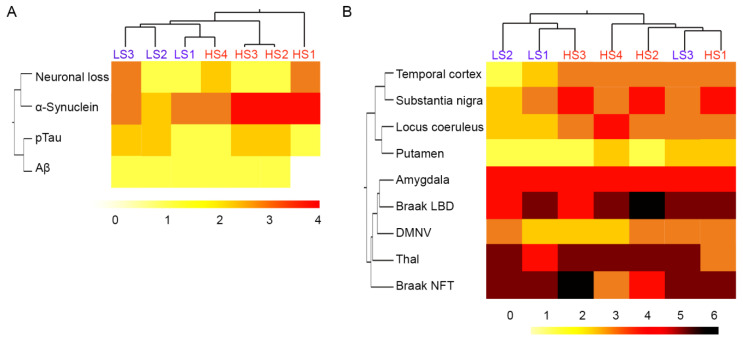
Cluster analysis of neuropathologic scores between α-synuclein high and low seeders. Neuropathologic scores of neuronal loss, α-synuclein, phosphorylated tau, and Aβ in the substantia nigra (**A**). α-Synuclein semiquantitative scores across the brain, Braak NFT and LBD stages, and Thal phases (**B**). The darker color represents the higher semiquantitative scores. Abbreviations: Aβ: amyloid beta; DMNV: dorsal motor nucleus of the vagus; HS: high seeder; LS: low seeder; NFT: neurofibrillary tangle; pTau: phosphorylated tau.

Consistent with our previous findings [8], semiquantitative neuropathologic scores for neuronal loss, α-syn, tau, and Aβ pathologies in the SN did not distinguish between α-syn high and low seeders in unsupervised cluster analysis (Figure 1A). The representative figures are provided in Supplementary Figure S1. Similarly, semiquantitative α-syn scores across the brain, Braak LBD stages, Thal Aβ phases, and Braak NFT stages did not distinguish cases between α-syn high and low seeders (Figure 1B). As we reported previously [9], the 5G4 α-syn antibody revealed more α-syn pathology compared to the phosphorylated-α-syn antibody. However, the clustering pattern remained consistent with both antibodies.

### 2.4. RNA Quality

The DV200 values, assessing the proportion of fragments exceeding 200 nucleotides and recognized as a crucial indicator of RNA quality, consistently surpassed the 66.1% threshold established by Nagakubo et al. [21] across all our samples (Table 1). Moreover, the nSolver software (version 4, nanoString Technologies Inc., Seattle, WA, USA) did not flag any samples for suboptimal quality. These findings confirm the adequacy of RNA quality in our study samples. Genes with counts below the background level were filtered out using the nCounter Advanced Analysis software (version 2, nanoString Technologies Inc.), leaving 1811 genes for subsequent analysis (Supplementary Table S1).

### 2.5. Gene Expression Analysis

**Figure 2:**
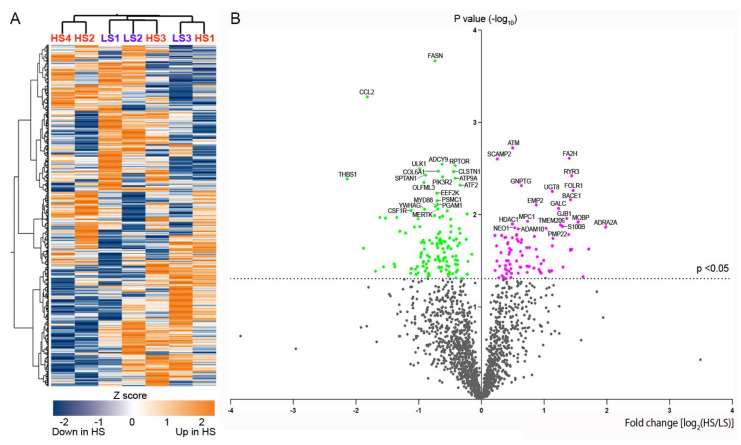
Heatmap, cluster analysis (**A**), and volcano plot (**B**) of the genes studied. Purple represents upregulated and green demonstrates downregulated genes in α-syn high seeders with unadjusted *p*-value < 0.05. Abbreviations: HS: high seeder; LS: low seeder.

**Figure 3:**
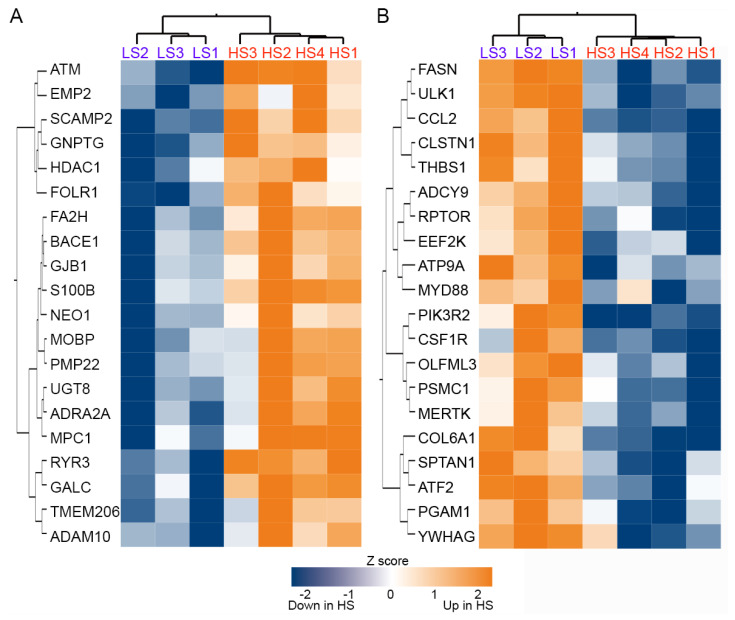
Heatmap and cluster analysis of the top 20 upregulated (**A**) and downregulated (**B**) genes in α-syn high seeders compared with low seeders. Abbreviations: HS: high seeder; LS: low seeder.

Unsupervised cluster analysis of the expression level of 1811 genes in the SN did not differentiate between the α-syn high and low seeders (Figure 2A). Although none of the genes exhibited an adjusted *p*-value < 0.05 for comparison between high and low seeder groups, 223 genes had an unadjusted *p*-value < 0.05 from the set of 1811 (Figure 2B). Heatmaps depicting the top 20 upregulated and downregulated genes in high seeders versus low seeders are presented in Figure 3A (upregulated genes) and Figure 3B (downregulated genes), with detailed lists in Table 2 (upregulated genes) and Table 3 (downregulated genes). Among the top 20 upregulated genes, 5 are related to membrane functions: *SCAMP2*, *RYR3*, *UGT8*, *ADRA2A*, and *ADAM10*. Additionally, two genes, *ATM* and *UGT8*, are associated with lipid metabolism. In the top 20 downregulated genes, 6 are linked to membrane activities: *CCL2*, *COL6A1*, *CLSTN1*, *PIK3R2*, *ATP9A*, and *ATF2*. *ATF2* is further implicated in lipid metabolism, while another gene, *PSMC1*, is specifically linked to the UPS. *SNCA* (synuclein alpha) exhibited downregulation in high seeders compared to low seeders (fold change −1.3, *p* = 0.0015). Transcripts associated with dopaminergic neurons such as *TH* (Tyrosine hydrolase, fold change −4.33, *p* = 0.265), *SLC6A3* (sodium-dependent dopamine transporter, fold change −0.849), and *NR4A2* (nuclear receptor related 1, fold change −0.375, *p* = 0.61), showed downregulation in high seeders. On the contrary, transcripts related to glial activation, including *S100B* (fold change 1.29, *p* = 0.013), *GFAP* (fold change 0.252, *p* = 0.537), and *ALDH1L1* (fold change 0.648, *p* = 0.537), were upregulated in high seeders.

**Figure 4:**
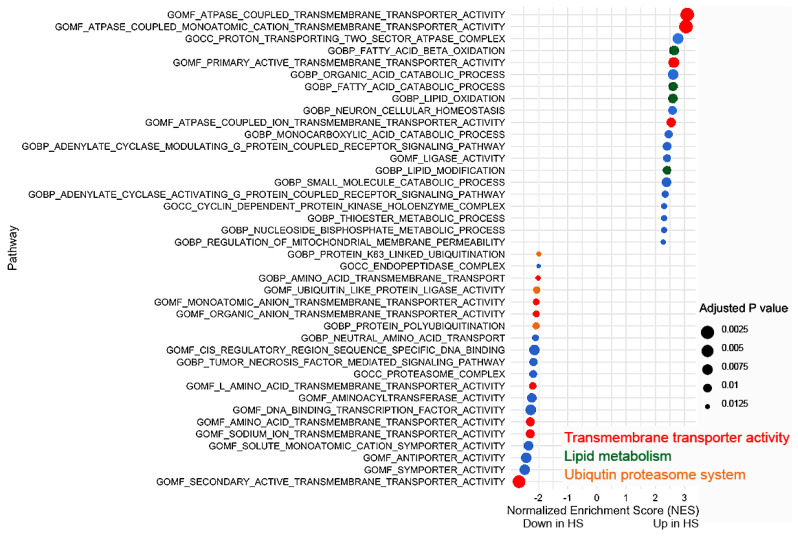
GSEA of the top 20 upregulated (upper part of the gene list) and downregulated (lower part of the gene list) pathways in α-syn high seeders compared with low seeders. Red represents transmembrane transporter activity, green demonstrates lipid metabolism, orange exhibits ubiquitin proteasome system pathways, and blue indicates other pathways. Abbreviation: BP: biological process; CC: cellular component; GO: gene ontology; HS: high seeder; MF: molecular function.

We then proceeded for gene set enrichment analysis (GSEA) and compared high with low seeders. This analysis highlighted 112 pathways with an adjusted *p*-value < 0.05 out of 3819 pathways in the C5 gene set. The leading 20 upregulated and downregulated pathways from the GSEA are depicted in Figure 4. Among the altered pathways in α-syn high seeders, four transmembrane transporter activity pathways were upregulated and seven were downregulated (Figure 4). Notably, a pathway related to mitochondrial membrane permeability regulation was significantly enhanced in α-syn high seeders. Additionally, four lipid metabolism pathways were upregulated, while three ubiquitination-related pathways showed downregulation in these seeders. Comprehensive pathway details, including *p*-values, NES, size, and leading-edge genes, are provided in Supplementary Table S2.

## 3. Discussion

This study revealed the association of molecular behavior of α-syn in the SN with membrane transports, lipid metabolism, and ubiquitin–proteasome system in LBD.

Using nanoString nCounter technology, we analyzed 1811 mRNAs, which facilitates direct mRNA expression measurement with a small sample across numerous genes without necessitating cDNA conversion or polymerase chain reaction [22]. While no individual genes demonstrated an adjusted *p*-value < 0.05, we identified the top 20 upregulated and downregulated genes and pathways when comparing α-syn high seeders versus low seeders in the SN.

Accumulated evidence suggests that misfolded α-syn interferes with vital cellular function and overwhelms the cellular protein degradation system. In our previous studies, we observed a marked enrichment of proteins associated with organelle inner and outer membranes, as well as components of the extrinsic membrane in the SN exhibiting high α-syn seeding activity [8]. Consistent with this observation, the most markedly differentially expressed genes and pathways in the current study were associated with transmembrane transporters. Specifically, among the top 20 upregulated genes and pathways, five genes and four pathways associated with transmembrane transports were upregulated. In contrast, six genes and seven pathways linked to transmembrane transports were downregulated in α-syn high seeders relative to their low-seeding counterparts. Although it remains to be fully determined how α-syn crosses the cellular and organelle membranes, several possibilities have been postulated such as direct penetration [23], annular pore-like structures [24], tunneling-nanotubes [25], and endocytosis [26]. Membranes, acting as barriers, regulate solute concentrations in adjacent aqueous compartments inside and outside [27]. Transmembrane transport is controlled by complex interactions between membrane lipids, proteins, and carbohydrates [27]. The basic types of membrane transport are simple passive diffusion (by channels and carriers) and active transport [27]. Passive diffusion requires no additional energy source while active transport requires additional energy, often in the form of ATP [27]. *ATP13A2* (PARK9), a gene associated with a levodopa responsive form of parkinsonism, that is a member of the *p*-type ATPase transporter, involves α-syn externalization through exosomes [28,29]. Our study revealed three upregulated pathways associated with ATPase-coupled transmembrane transporters, suggesting a potential role in heightened α-syn externalization in α-syn high seeders.

Transmembrane transports also encompass both endocytosis and exocytosis [27]. These membrane trafficking mechanisms have received considerable attention owing to their potential roles as initiators or enhancers of the neurodegenerative processes leading to LBD [30]. α-Syn is a membrane-binding protein with a number of possible normal functions including control of synaptic membrane processes and biogenesis, regulation of neurotransmitter release, and synaptic plasticity [31,32,33,34,35,36]. However, overexpression of wild-type and mutated α-syn [35,37], oligomeric α-syn [34], and small (less than 200 nm) non-fibrillar α-syn [38] can cause loss of membrane integrity, thinning the membrane and/or the formation of pores in the cell membrane, leading to uncontrolled diffusion of molecules in and out of the cell [35]. Additionally, membrane thinning has been observed in other amyloidogenic proteins including Aβ [39]. Based on these observations, we postulate that disturbances in membrane homeostasis facilitate the mobility of α-syn, leading to increased propagation activity. Such mechanisms could potentially enhance the seeding activity of α-syn.

Two genes and four pathways associated with lipid metabolism and a pathway with mitochondrial membrane permeability were significantly upregulated in α-syn high seeders, consistent with our previous proteomics report [8]. Another study also highlighted the overexpression of the fatty acid beta-oxidation in the nigral proteome of PD [40]. Additionally, significant lipid accumulation has been documented in LBs [3,41,42]. The primary and most recognized role of lipids is in forming the basic structure of cell membranes [43]. The interaction of α-syn with lipid membranes is believed to drive its oligomerization and subsequent aggregation [44,45]. Oligomeric forms or small soluble non-fibrillar aggregates of α-syn can compromise lipid membrane integrity, leading to membrane permeabilization [35,38,46]. Collectively, these insights suggest that enhanced lipid metabolism may promote α-syn aggregation, fostering the formation of more toxic α-syn variants and accelerating α-syn seeding activity.

Moreover, one gene and three pathways linked to the UPS were found to be downregulated in α-syn high seeders. The pivotal role of UPS and the ALP in the neurodegeneration of LBD has been firmly established [12,47,48]. Both UPS and ALP serve as primary intracellular degradation mechanisms, particularly when cells are confronted with misfolded protein aggregates [47,48,49]. Dysfunction in UPS is recurrently implicated across various genetic etiologies of familial PD [47,50]. Notably, both the pale body, an early cytoplasmic alteration preceding LB formation [1,2,3,51], and LBs themselves contain ubiquitinated proteins alongside lysosomes [3,47,51,52]. Our prior proteomics analysis [8] also highlighted disruptions in lysosomal organization within α-syn high seeders. The accumulation of aggregated α-syn can impede ALP function, leading to compromised clearance and further synuclein accumulation [48,49]. A recent cellular model of α-syn propagation underscored that lysosomal membrane rupture facilitates the release and spread of accumulated α-syn [53]. Collectively, these insights indicate that compromised cellular degradation mechanisms in α-syn high seeders may hinder the breakdown of misfolded α-syn, thereby exacerbating its propagation.

In this study, we observed a downregulation of *SNCA* in high seeders compared to low seeders. Our previous findings [8] indicated that the total amount of nigral α-syn protein in the PBS-soluble fraction, as measured by ELISA, did not exhibit a link to α-syn seeding activity. However, a negative correlation was observed between the levels of aggregated synuclein protein and α-syn seeding activity. The impact of the decreased *SNCA* on the reduction in aggregated α-syn in high seeders has remained unclear. However, our recent study on neuron-specific *SNCA* expression showed gradual decrease during the development of Lewy bodies [54]. We speculate that the increased demand of *SNCA* expression to produce physiological α-syn protein for the seeding of the misfolded α-syn exhausts the mRNA production, which is more prominent in high seeders. Nevertheless, to elucidate the association between the expression levels of α-syn transcript, protein, and seeding activity, further investigation and validation are warranted.

In the present study, transcripts linked to dopaminergic phenotype exhibited downregulation, while those associated with glial activation displayed upregulation in high seeders. These observations imply that dopaminergic neuron loss and glial activation are more pronounced in high seeders compared to their low seeder counterparts in the molecular level.

The fundamental principle of molecular biology underscores that proteins are synthesized from mRNA templates [55]. Therefore, mRNA expression levels typically correlate with protein synthesis [55,56,57,58,59]. However, this correlation of expression levels between mRNA and protein varies widely and is imperfect [55,60], in which targeted proteomics across cell lines and tissues have reported r-values between 0.39 and 0.79 [55,61]. The intricate relationship between mRNA and protein expression remains enigmatic. For example, elevated mRNA levels could either signify enhanced protein expression or, result from a negative feedback mechanism due to reduced protein expression. Consequently, interpreting mRNA levels proves challenging, and it may be prudent to infer that both elevated and reduced mRNA expression levels signify a disturbance in homeostasis from the normal state. However, it is important to emphasize that our transcriptomics observations recapitulate our findings using proteomics [9] supporting the relevance of our findings.

As previously highlighted [8], routine neuropathological assessments did not discern or predict α-syn seeding activity. This emphasizes the potential molecular differences between high and low seeders, especially in light of the consistent neurodegeneration and protein deposits observed across all cases. Hence, transcriptomic and proteomic analyses offer valuable insights into α-syn high seeders’ characteristics.

Several study limitations warrant consideration. First, the sample size was small and underpowered, as indicated by the lack of statistical significance in the adjusted *p*-values, necessitating validation in larger cohorts. Second, we cannot definitively ascertain whether the observed alterations in the targeted transcriptome precede or result from the presence of distinct α-syn species. Third, the variability in post-mortem intervals among our cases could impact mRNA integrity, given the rapid degradation of mRNAs in human autopsy tissues post-mortem [62]. Nevertheless, the quality of our samples, as indicated by the DV200 values, remained adequate. The nanoString nCounter assay employed in this study can effectively analyze minute RNA quantities, directly enumerating individual RNA transcripts without necessitating additional enzymatic steps, amplification, or cDNA conversion [22]. Fourth, our LBD cases exhibited a confluence of pathologies, with intermediate to high levels of ADNC. It is plausible that concurrent Aβ and/or tau pathologies might influence the molecular profiles observed in our LBD cohort. However, since all cases contained some levels of Aβ and tau in the SN, it is highly plausible that the molecular behavior of α-syn is the major driving force of the transcriptomic and proteomic differences between low and high seeders. Fifth, non-diseased control cases were not included in our study. However, the primary objective of this investigation is to clarify the association between the molecular behavior of α-syn and transcriptomic changes in high and low seeders. While a large number of studies have previously explored differences between LBD and normal controls [10,11,12,13,14,15,16,17,18,19,20], the specific distinctions in transcriptomics related to seeding activity have not been reported.

## 4. Materials and Methods

### 4.1. Materials

We collected frozen brain tissues and 4 μm thick formalin-fixed paraffin-embedded sections from the University Health Network-Neurodegenerative Brain Collection (Toronto, ON, Canada) with confirmed neuropathological diagnoses. We carefully selected age, sex, seeding activity, and mixed pathology matched 7 LBD cases with our previously reported proteomics analysis [8], with 4 high α-syn seeders (HS 1—4) and 3 low seeders (LS 1—3). One high seeder (HS 1) and 2 low seeders (LS 2, 3) were included in our previous proteomics analysis [8]. Details on age at death, sex, disease duration, post-mortem intervals, and neuropathological examination are outlined in Table 1. This study received approval from the University Health Network Research Ethics Board and the University of Toronto (Nr. 20-5258 and 39459).

### 4.2. α-Syn SAA

α-Syn SAA was performed to investigate the seeding capacity of the misfolded α-syn present in the SN. This assay was conducted in 32 cases of neuropathologically confirmed LBD cases, as previously reported [8].

### 4.3. Neuropathologic Analysis

Routine histological examination and immunohistochemistry (IHC) were performed on 4 μm formalin-fixed paraffin-embedded tissue sections, using the following antibodies: Aβ (6F/3D, 1:50, Dako, Glostrup, Denmark), phosphorylated-tau (AT8, 1:1000, Thermo Fischer, Waltham, MA, USA), disease-associated α-syn (5G4, 1:4000, Analytikjena, Jena, Germany), and phosphorylated α-syn (clone #64, 1:10,000, FUJIFILIM Wako Pure Chemical Corporation, Osaka, Japan). Antigen retrieval was carried out using Dako PT Link with a low pH solution, except for the anti-Aβ antibody. For the anti-Aβ antibody, where 80% formic acid was applied for 1 h, and for the 5G4 antibody, it was applied for 5 min. According to the manufacturer’s protocol, immunostaining was performed using Dako Autostainer Link 48 and EnVision FLEX+ Visualization System. Subsequently, all sections were counterstained with hematoxylin. All cases had a standard neuropathological assessment based on the current consensus criteria including Braak LBD stage [63], Lewy pathology consensus criteria [64], and National Institute of Aging-Alzheimer’s Association (NIA-AA) Alzheimer’s disease neuropathological change (ADNC) [65,66,67]. In addition to the staging system described above, we evaluated the severity of neuronal loss, Lewy pathology, tau pathology, and Aβ plaques using a semi-quantitative five-point scoring system: Score 0 indicated no pathology, Score 1 denoted minimal, Score 2 reflected mild, Score 3 implied moderate, and Score 4 signified severe [68,69,70].

### 4.4. RNA Extraction

Frozen human brain tissue of the SN was micro-dissected as previously described [8,9,71]. The brain tissue was subjected to dissociation using the gentleMACS Octo Dissociator (Miltenyi Biotec, Bergisch Gladbach, Germany), after which RNA extraction was carried out utilizing the Qiagen Lipid Tissue Mini Kit (Qiagen, Venlo, The Netherlands), as directed by the manufacturer’s protocol. Sample concentration, purity, RNA quality, and fragmentation were assessed using the Nanodrop ND-1000 Spectrophotometer (ThermoFisher) and Bioanalyzer (2100 BioA, Agilent, Santa Clara, CA, USA), respectively. For gene expression analysis, 100 ng of RNA was utilized from each sample. Gene expression profiling employed nanoString nCounter panels (nanoString Technologies Inc.) covering neuropathology, neuroinflammation, glial profiling, and metabolic pathways.

### 4.5. Data Analysis and Statistics

The mRNA count data were normalized by the default settings of nSolver Analysis software. Subsequent analysis for fold changes and *p*-values, comparing gene expression in α-syn high seeders to low seeders, was conducted using the nCounter Advanced Analysis software. Pathway analysis utilized the RStudio package (version 2023.12.0+369) [72], incorporating the C5 ontology gene sets from the Human Molecular Signatures Database v2023.2.Hs [73,74]. Welch’s *t*-test, based on gene expression Z-scores, was applied to the high and low seeder groups. Genes were ranked according to the t-statistic. Gene set enrichment analysis employed the fgsea package (version 1.26.0) [75], with results sorted by the normalized enrichment score (NES). Neuropathology and transcriptomic data underwent cluster analysis using JMP 14.3 software (JMP Statistical Discovery LLC, Cary, NC, USA). Visualizations for gene analyses were generated using Graphpad Prism (v.10, Graphpad Software Inc., San Diego, CA, USA), JMP, and ggplot2 package in R (version 3.4.4) [76]. Fisher’s exact test or Mann–Whitney U test was applied to compare the demographic data between α-syn high and low seeders using SPSS software (v25, IBM, Chicago, IL, USA). To address multiple comparisons, the Benjamini–Hochberg method adjusted *p*-values to estimate false discovery rates in gene expression data. A significance threshold of *p* < 0.05 was applied using a two-tailed test.

## 5. Conclusions

In conclusion, our findings elucidate the intricate molecular signatures associated with α-syn high seeders. We emphasize the disruptions in membrane transporters, lipid metabolism, and the UPS as potential contributors to enhanced α-syn seeding activity. This study presents a novel perspective proposing that strategies aimed at modulating these pathways could progress innovative future precision medicine for patients with LBD, a complex and heterogeneous disease, stratified based on the distinct molecular behavior of α-syn.

## Figures and Tables

**Table 1:** Demographic data and neuropathological assessments of the cohort.

Case	Age at Death	Sex	Disease Duration, Year	PMI, Hour	Clinical Phenotypes	Pathological Diagnosis	Braak LBD Stage	NIA-AA ADNC	DV200, %
HS1	73	M	8	9	DLB	LBD	5	Intermediate, A2B3C2	90
HS2	79	M	6	8.5	DLB	LBD	6	Intermediate, A3B2C2	86
HS3	80	M	7	5	DLB	LBD	4	High, A3B3C2	88
HS4	92	F	10	NA	DLB	LBD	5	Intermediate, A3B2C1	86
LS1	59	F	1 *	12.5	DLB	LBD	5	High, A3B3C3	73
LS2	65	F	12	3	DLB	LBD	4	High, A3B3C3	85
LS3	72	M	7	6.5	DLB	LBD	5	High, A3B3C3	88

Note: *, The cause of death: a car accident. Abbreviations: DLB, dementia with Lewy body; HS, α-syn high seeder; LS, α-syn low seeder; NA, not available; NIA-AA ADNC, National Institute of Aging-Alzheimer’s Association Alzheimer’s disease neuropathological change; PMI, post-mortem interval.

**Table 2:** Top 20 upregulated genes in α-syn high seeders.

Gene Symbol	Official Full Name	Representative Pathways in C5 Gene Ontology Set	Reports in LBD or α-Syn	Fold Change (log2)	*p*-Value	Adjusted *p*-Value
*ATM*	ATM serine/threonine kinase	Lipid metabolic process, cell cycle phase transition	None	0.498	0.0019	0.374
*FA2H*	Fatty acid 2-hydroxylase	Schwann cell differentiation	None	1.4	0.0025	0.374
*SCAMP2*	Secretory carrier membrane protein 2	Recycling endosome membrane, Golgi vesicle transport	None	0.253	0.0025	0.374
*RYR3*	Ryanodine receptor 3	Calcium channel activity, transmembrane transporter activity	Association with neuronal calcium dyshomeostasis related to RyR and PD development [22].	1.44	0.0038	0.416
*GNPTG*	N-acetylglucosamine-1-phosphate transferase subunit gamma	None	None	0.636	0.0048	0.46
*FOLR1*	Folate receptor alpha	Cell recognition	None	1.46	0.0055	0.46
*UGT8*	UDP glycosyltransferase 8	Neuron development, membrane lipid metabolic process	None	1.13	0.0056	0.46
*BACE1*	Beta-secretase 1	Neuron apoptotic process, presynapse	BACE1 mRNA increase in the superior frontal gyrus in PD/DLB brains [23]. *BACE1* polymorphism increases the risk of PD [24].	1.42	0.0069	0.46
*EMP2*	Epithelial membrane protein 2	Caveola	None	0.873	0.0078	0.501
*GALC*	Galactosylceramidase	Abnormal head blood vessel morphology	Association with silent *GALC* mutations and the development of PD [25].	1.23	0.0086	0.501
*GJB1*	Gap junction protein beta 1	None	None	1.36	0.011	0.501
*MPC1*	Mitochondrial pyruvate carrier 1	Abnormal circulating carbohydrate concentration	MPC inhibition is neuroprotective in multiple neurotoxin-based and genetic models of PD [26].	0.737	0.0118	0.501
*MOBP*	Myelin-associated oligodendrocyte basic protein	None	MOBP polymorphism is a risk factor for PD [27]. MOBP immunoreactivity in LB [28].	1.54	0.012	0.501
*HDAC1*	Histone deacetylase 1	None	HDAC1 inhibitor alleviates neuronal death in the experimental PD models [29].	0.492	0.0126	0.501
*TMEM206*	Transmembrane protein 206	None	None	1.26	0.0129	0.501
*S100B*	S100 calcium binding protein B	Regulation of neuron differentiation, regulation of synaptic plasticity	Association with S100B polymorphisms and PD onset age [30]. S100B protein was higher in the SN of PD [31].	1.29	0.0134	0.501
*ADRA2A*	Adrenoceptor alpha 2A	Adenylate cyclase activating G protein-coupled receptor signaling, transmembrane transport	α-2-adrenergic receptor binding increase in the locus coeruleus projection areas in DLB [32].	1.98	0.0136	0.501
*NEO1*	Neogenin 1	Axon guidance	None	0.532	0.0139	0.501
*PMP22*	Peripheral myelin protein 22	Sensorimotor neuropathy	None	1.03	0.014	0.501
*ADAM10*	ADAM metallopeptidase domain 10	Synapse, plasma membrane region	*ADAM10* polymorphism increases the risk of PD [33].	0.585	0.0143	0.501

**Table 3:** Top 20 downregulated genes in α-syn high seeders.

Gene Symbol	Official Full Name	Representative Pathways in C5 Gene Ontology Set	Reports in LBD or α-Syn	Fold Change (log2)	*p*-Value	Adjusted *p*-Value
*FASN*	Fatty acid synthase	Oxidoreductase activity	None	−0.74	0.00022	0.347
*CCL2*	C-C motif chemokine ligand 2	Humoral immune response, positive regulation of transmembrane transport	CCL2 promotes α-syn secretion and the neuronal apoptosis induced by α-syn [34]. Correlation with CCL2 and PD clinical stage and autonomic symptom [35].	−1.82	0.00053	0.347
*ADCY9*	Adenylate cyclase 9	Adenylate cyclase modulating G protein-coupled receptor signaling	None	−0.626	0.00284	0.374
*RPTOR*	Regulatory-associated protein of MTOR complex 1	Regulation of cell development, negative regulation of autophagy	Association of *RPTOR* with *SNCA* and differential age at onset in PD [36].	−0.417	0.00293	0.383
*ULK1*	Unc-51-like autophagy activating kinase 1	Cellular response to stress, process utilizing autophagic mechanism	ULK1 increase in LRRK2 PD brains [37]. ULK1 immunoreactivity in LB [38]. ULK1 protein increase in PD blood mononuclear cells [39].	−0.984	0.00319	0.383
*COL6A1*	Collagen type VI alpha 1 chain	Vacuolar membrane, collagen biding	None	−0.686	0.00338	0.416
*CLSTN1*	Calsyntenin 1	Postsynaptic membrane, vesicle-mediated transport	CLSTN1 increase in CSF in DLB [40].	−0.445	0.00341	0.416
*SPTAN1*	Spectrin alpha, non-erythrocytic 1	Negative regulation of cytoskeleton organization	None	−0.89	0.00373	0.416
*PIK3R2*	Phosphoinositide-3-kinase regulatory subunit 2	Phosphatidylinositol metabolic process, extrinsic component of membrane	Association with rare variants of *PIK3R2* and DLB [41].	−0.619	0.00389	0.416
*ATP9A*	ATPase phospholipid transporting 9A	ATP dependent activity, endosome membrane	None	−0.418	0.00402	0.416
*THBS1*	Thrombospondin 1	None	*LRRK2* mutation promotes ER stress via interacting with THBS1/TGF-β1 in PD [42].	−2.14	0.0041	0.416
*OLFML3*	Olfactomedin-like 3	None	None	−0.918	0.00448	0.416
*ATF2*	Activating transcription factor 2	Regulation of mitochondrial membrane permeability, cellular lipid metabolic process	ATF2 contributes to dopaminergic neurodegeneration in the MPTP mouse model of PD [43].	−0.341	0.00478	0.46
*EEF2K*	Eukaryotic elongation factor 2 kinase	Postsynapse, regulation of dendritic spine morphogenesis	EEF2K increase in PD brain and EEF3K inhibition reduces α-syn toxicity [44].	−0.711	0.00587	0.46
*PSMC1*	Proteasome 26S subunit, ATPase 1	Endopeptidase complex, proteasome complex	Depletion of 26S proteasomes in mouse brain causes neurodegeneration and Lewy-like inclusions [45].	−0.708	0.00707	0.406
*PGAM1*	Phosphoglycerate mutase 1	Glycolytic process	None	−0.699	0.00776	0.501
*MYD88*	MYD88 innate immune signal transduction adaptor	Stress-activated protein kinase signaling cascade, toll-like receptor 4 signaling	TLR2/MyD88/NF-κB pathway reduces α-syn spreading [46].	−0.731	0.00813	0.501
*MERTK*	MER proto-oncogene, tyrosine kinase	None	MERTK mediates α-syn fibril uptake in microglia [47].	−0.692	0.00868	0.501
*YWHAG*	Tyrosine 3-monooxygenase/tryptophan 5-monooxygenase activation protein gamma	Cellular response to insulin stimulus, phosphoprotein binding	YWHAG increase in the SN in PD [48].	−0.905	0.00876	0.501
*CSF1R*	Colony stimulating factor 1 receptor	Neurotransmitter secretion, regulation of leukocyte migration	CSF1R mutation in a case presenting as DLB [49].	−1.12	0.00899	0.501

Abbreviations: ER: endoplasmic reticulum; LRRK2: leucine-rich repeat serine/threonine-protein kinase 2; MPTP: 1-methyl-4-phenyl-1,2,3,6-tetrahydropyridine; NF-κB: nuclear factor kappa-light-chain-enhancer of activated B-cells; *SNCA*: synuclein alpha; TLR: Toll-like receptor.

## Data Availability

The datasets used and analyzed during the current study are available from the corresponding author upon reasonable request.

## Supplementary Materials

The following supporting information can be downloaded at: https://www.mdpi.com/article/10.3390/ijms25052676/s1.
